# A Mini-Review of the Role of Glutamate Transporter in Drug Addiction

**DOI:** 10.3389/fneur.2019.01123

**Published:** 2019-10-22

**Authors:** Wenjun Wang, Fancai Zeng, Yingying Hu, Xiang Li

**Affiliations:** ^1^Institute for Cancer Medicine and School of Basic Medical Sciences, Southwest Medical University, Luzhou, China; ^2^Department of Biochemistry and Molecular Biology, School of Basic Medical Science, Southwest Medical University, Luzhou, China

**Keywords:** addiction, glutamate transporters, excitatory amino acid transporters, vesicular glutamate transporters, glutamate

## Abstract

**Goals:** The development of new treatment for drug abuse requires identification of targetable molecular mechanisms. The pathology of glutamate neurotransmission system in the brain reward circuit is related to the relapse of multiple drugs. Glutamate transporter regulates glutamate signaling by removing excess glutamate from the synapse. And the mechanisms between glutamate transporter and drug addiction are still unclear.

**Methods:** A systematic review of the literature searched in Pubmed and reporting drug addiction in relation to glutamate transporter. Studies were screened by title, abstract, and full text.

**Results:** This review is to highlight the effects of drug addiction on glutamate transporter and glutamate uptake, and targeting glutamate transporter as an addictive drug addiction treatment. We focus on the roles of glutamate transporter in different brain regions in drug addiction. More importantly, we suggest the functional roles of glutamate transporter may prove beneficial in the treatment of drug addiction.

**Conclusion:** Overall, understanding how glutamate transporter impacts central nervous system may provide a new insight for treatment of drug addiction.

## Introduction

**Figure 1:**
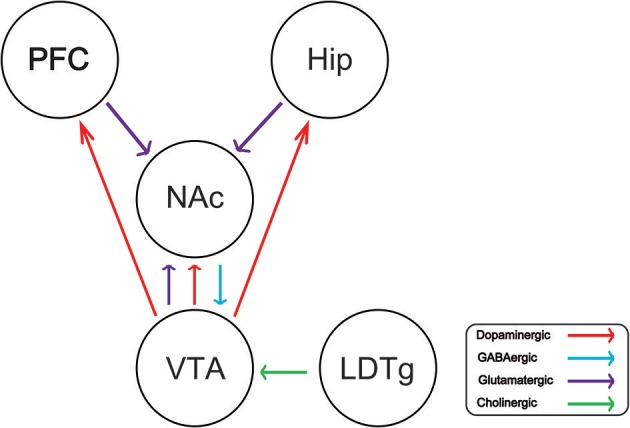
Schematic of brain reward circuitry implicated in addiction. The ventral tegmental area (VTA) projects dopaminergic (red) transmission to the nucleus accumbens (NAc), hippocampus (Hip), prefrontal cortex (PFC). The NAc receives Glutamatergic (purple) inputs from the PFC, VTA, and Hip. The VTA receives GABAergic (blue) from the NAc. The VTA receives cholinergic (green) input from the laterodorsal tegmental nucleus (LDTg).

Drug addiction is a chronic and recurrent mental disorder characterized by compulsive and uncontrollable drug use and addiction behavior (1). There is growing evidence that drug abuse-induced changes in synaptic plasticity, especially in the midbrain dopamine system, lead to long-term effects and contribute to relapse after withdrawal (2). Drug addiction can also inhibit the central respiratory system and reduce the sensitivity of the respiratory center to carbon dioxide. Long-term use of addictive drugs can lead to pathological changes in the related brain areas and produce related pathological behavior, such as drug seeking, drug withdraw, and relapse. The molecular mechanisms of drug addiction are mainly involved in the following four brain regions: prefrontal cortex (PFC), ventral ventral tegmental area (VTA), nucleus accumbens (NAc), and hippocampus (Hip). After drug addiction, the pathological behavior and memory are closely related to these brain regions. In addition, the most important thing is that the pathological changes in these brain regions will change the neural projection of the brain and the synaptic plasticity of the neurons. The addiction of some drugs limited its clinical implication. For example, morphine is the first-line choice for the management of chronic pain in both cancer or non-cancer patients (3–5). Dolantin is a synthetic opioid receptor agonist. Although dolantin has been used in the clinical treatment of pain instead of morphine, the analgesic effect of dolantin is about 10 percent of morphine. Beside that, chronic administration of dolantin also leads to addiction and tolerance. Therefore, it is necessary to investigate the mechanism of drug addiction. Recently, lots of studies have provided evidence for the complexity of anatomical and functional interactions between neuros in brain reward circuits prompted by drug's rewarding action, including dopaminergic neurons, glutamatergic neurons, and gama aminobutyric acid neurons. Recently research has reported that glutamatergic neurons is closely related to drug addiction, because it is involved in learning association in mesocorticolimbic reward circuitry. The PFC glutamatergic neurons projection to the NAc plays an important role in drug seeking behavior (6). NAc also receives glutamatergic input from Hip and VTA, which has been proved that this circuit is implicated in drug addiction (7–9). Cholinergic neurons from laterodorsal tegmental nucleus (LDTg) activate dopamine neurons in the reward circuit via projecting to the VTA (10). GABAergic neurons from NAc project to the VTA associating with rewarding by regulating DA neurons activity (11). Schematic of brain reward circuitry was shown in Figure 1. However, there is less research on glutamate transporters in drug addiction. Here, we review the glutamate transporters in brain reward circuits under drug addiction.

Glutamate transporters (GLT) play an important role in physiological glutamate homeostasis, neurotoxicity, and glutamatergic regulation of opioid tolerance (12–16). It has been found that there are two kinds of glutamate transporter, including vesicular glutamate transporters (VGLUTs) and excitatory amino acid transporters (EAATs) (17). Extracellular glutamate levels are regulated by high-affinity EAATs (18). EAATs are known to be responsible for maintaining the homeostasis of the extracellular glutamate concentration by protecting neurons against detrimental overstimulation of glutamatergic receptors (19). EAATs are classified into five different subtypes: glutamate aspartate transporter (EAAT1), glutamate transporter-1 (EAAT2), excitatory amino acid carrier-1 (EAAT3), EAAT4, and EAAT5 (20). In addition to the aforementioned mechanisms, glutamate concentrations are also regulated by modulating glutamate internalization into synaptic vesicles through VGLUTs 1, 2, and 3. The release of glutamate in the presynaptic area depends upon the expression and the function of secretory vesicles, vesicular glutamate transporters (VGLUTs). VGLUT family presents distinct expression patterns. VGLUT1 and VGLUT2 are the major secretory vesicles in the brain, and VGLUT3 often acts as a cotransporter of glutamate and other neurotransmitters, such as serotonin, gamma-aminobutyric acid (GABA), and acetylcholine (21). Tables 1, 2 provide a summary of the distribution of glutamate transporters and potential drug targets.

**Table 1:** Distribution of vesicular glutamate transporters.

**Types of VGLUT**	**Majority distribution**	**Location**	**Target for addiction**	**References**
VGLUT1	Neocortex and hippocampus (22) Spinal cord (23) Hypothalamus and amygdala (24) Prefrontal cortex (25) Nucleus accumbens (26) Striatum (27) Cerebellum (28)	Synaptic vesicles	Potential	(29)
VGLUT2	Ventral ventral tegmental area (30) Basolateral amygdala (31) Nucleus accumbens (32)	Synaptic vesicles	Yes	(33)
VGLUT3	Caudate-putamen, olfactory tubercle Nucleus accumbens Hippocampus Interpeduncular nucleus and dorsal Medial raphe nuclei (34)	Synaptic vesicles	Yes	(35)

**Table 2:** Distribution of excitatory amino acid transporters.

**Types of VGLUT**	**Majority distribution**	**Cell-type**	**Target for addiction**	**References**
EAAT1	Cerebellum (36) Cortex (17) Spinal cord (37)	Glial cells	Potential	(38)
EAAT2	Whole brain (17) Spinal cord (37)	Glial cells	Potential	(39)
EAAT3	Hippocampus Cerebellum Striatum (40)	Neuron	Yes	(41)
EAAT4	Cerebellum (40)	Neuron	Unknown	–
EAAT5	Retina (42)	Photoreceptors Bipolar cells	Unknown	–

## Methods

This review is according to literature study in Pubmed until March 31st 2019. Pubmed was searched by using free-text terms and addiction subject heading. A uniform search strategy was applied to Pubmed to identify the reported studies. The primary and keywords were as following: addiction, withdrawal, relapse, reward VGLUT1, VGLUT2, VGLUT3, EAAT1, EAAT2, EAAT3, EAAT4, EAAT5, PFC, VTA, NAc, Hip, GLP, Glu, glutamate neuron, dopamine neuron, cholinergic interneurons, hippocampal neurons and neuroplasticity. All the studies were screened by title, abstract, and full text.

### The Role of GLT in Addiction in the VTA

The VTA is a tiny area near the midbrain, which is involved reward effects (43–46). The VTA mainly contains three types of neurons: dopamine neurons make up about 60–65% of the cells in the VTA, GABAergic neurons make up ~30–35% of the cells in the VTA, a population of glutamate neurons make up ~2–3% of the cells in the VTA (7). Yamaguchi et al. have proved that VGLUT2 mRNA but not VGLUT1 mRNA was expressed in the VTA (30). VTA glutamatergic neurons–expressing vesicular glutamate transporter2 (VGLUT2)–project to limbic and cortical regions, but also excite neighboring dopaminergic neurons (47). VGLUT2 was also found in dopaminergic neurons from VTA, which projects to NAc (30, 48). VGLUT2 exists in these neurons and allows glutamate to release from VTA dopaminergic neurons (49). This synergistic effect between glutamate and dopamine signaling may be important for the plasticity of postsynaptic AMPA receptors (50). Therefore, VTA plays an important role in drug addiction. Wang et al. suggested that photoactivation of VTA VGLUT2 neurons expressing Channelrhodopsin-2 (ChR2) under VGLUT2 promoter causes conditioned place preferences and also reinforces instrumental behavior. They also found that activation of VTA VGLUT2 neurons is mediated by local AMPAR and NMDAR. In addition, VTA VGLUT2 neurons mediate the development of place preference by releasing glutamate into the VTA, resulting in activation of both NMDA and AMPA receptors (47). The loss of VGLUT2 expression in DA (dopamine) neurons in VTA probably leads to a decrease in excitatory activity of the affected dopaminergic neurons. VTA DA neurons mediate the rewarding effects of psychostimulants such as amphetamine by increasing the level of extracellular DA in limbic areas such as the NAc (51). Birgner et al. proved that DAT-Cre/Vglut2Lox mice attenuated behavioral response to amphetamine compared to the control mice (52). These findings suggested that VGLUT2 played an important role for mediating rewarding effects of drugs of addiction. Behavioral studies have proved that optogenetic activation of VTA VGLUT2 neurons or their axonal terminals elicit aversive (47, 53–55). Until now, the role of some other glutamate transporters in addiction has been poorly investigated. Therefore, it is necessary to detect the role of glutamate transporters in addiction.

### The Role of GLT in Addiction in the NAc

The NAc is mainly composed of gamma-aminobutyric acid neurons. In addition, there are also Astrocyte cells and various types of Interneuron. The glutamate input to NAc mainly comes from the prefrontal cortex, thalamus, amygdala, and hippocampus. Different glutamate projections lead to different synapses and behavioral functions. The structure of NAc is complex, which can be divided into nucleus and shell regions according to its anatomical structure and the effects of reward (56–58). Medium spiny neurons (MSNs) from the NAc receive excitatory glutamatergic inputs and modulatory dopaminergic and cholinergic inputs from a variety of cortical and subcortical structures. The interaction between hippocampus and PFC glutamate input is thought to provide synaptic plasticity in MSNs to regulate reward learning (59, 60). Although cholinergic neurons are a minority group of NAc neurons, their projection in MSN has been shown to control drug addiction (61–63). In the NAc, VGLUT3 participates in the cooperative release of glutamate from these cholinergic neurons (64, 65). VGLUT1 is mainly expressed in the cortical structure, and it has been shown that NAc receives glutamate input from PFC and hippocampus (66). The accumulation of glutamate in the presynaptic membrane is mainly through the VGLUTS. The VGLUTS family controls the release of glutamate by the presynaptic membrane of the neuron (67, 68). The expression of VGLUTS is closely related to the level of glutamate in the synaptic cleft. In particular, VGLUT3 is expressed on cholinergic intermediate neurons in NAc, which plays an important role in the function of NAc (10, 69). Because of the different expression patterns of VGLUTs, these proteins can be used as presynaptic markers to understand the input of glutamate into NAc. Tukey et al. suggested that chronic uptake of sucrose did not change the expression of VGLUT1 in the synaptoneurosomes of NAc. Repeated intake of sucrose resulted in an increase in the level of VGLUT2 and VGLUT3 subunits in the synaptoneurosomes of NAc (70). Another study proved that silencing VGLUT3 in mice resulted in cocaine induced locomotor activity significantly (71). In addition, Sakae et al. showed that knocking out VGLUT3 increased cocaine addiction by increasing the glutamate signals of the NAc (35). Thus, in the model of addiction, VGLUTS state a new signal form of synaptic plasticity in the NAc.

### The Role of GLT in Addiction in the PFC

Projection neurons in the prefrontal cortex can regulate subcortical tissue structure, including ventral striatum and thalamus. Therefore, it can regulate the effects of addiction (72). Pyramidal neurons in the medial prefrontal cortex (mPFC) can receive nerves projections from different brain regions, including the basolateral amygdala. And at the same time, projection of glutamatergic neuron in the mPFC can also deliver to the VTA and NAc (73–75). In the limbic nervous system, including the prefrontal cortex, euphoria is associated with glutamate neurotransmission and the number of astrocytes. It is obvious that astrocytes regulate glutamate levels by removing glutamate from synapses by glutamate transporters. Glutamate neurons are mainly located in the prefrontal cortex. Studies have shown that the projection of glutamate neurons in the prefrontal cortex to the nucleus accumbens is an important rewarding pathway (76). VGLUT1 is mainly expressed in modulatory synapses, including PFC glutamate neurons project to the different brain regions of the reward circuit. Glutamate is the main driver of PFC neurons, and relapse to cocaine seeking requires the release of glutamate from the PFC projection to NAc (77). Glutamate transporter 1 (GLT-1) is responsible for the uptake of the majority of extracellular glutamate concentration (78, 79). Sari *et al*. showed that by upregulating the expression of glutamate transporter 1 blunts cue-induced reinstatement of cocaine-seeking behavior in rats (80). Their results suggested that glutamate played an important role in cue-induced relapse to cocaine-seeking behavior, implicating glutamate transporter 1 as a potential therapeutic target for cocaine addiction. Upregulating glutamate transporter 1 expression in mesocorticolimbic brain regions may serve as a potential treatment of drug addiction. The transmission of glutamate in synapse affects the excitability of neurons and the emotion. Drug abuse can lead to mood disorder. Therefore, to study the important role of glutamate transporter in the PFC is necessary and glutamate transporter may be a target to treat addiction.

### The Role of GLT in Addiction in the Hip

Hippocampus is an important brain tissue related to information storage, which has many kinds of functions. The most important function of hippocampus is to store memory information and learning ability. Therefore, the synaptic plasticity of hippocampal neurons is considered to be related to learning and memory. Vesicular glutamate transporters (VGLUTs) play an important role in synaptic function by uptake of glutamate in vesicles at the presynaptic terminal (81, 82). VGLUT1 and VGLUT2 are a vesicular glutamate transporter commonly, which are found in the telencephalic region, such as hippocampus. Beside that, some studies has proved that VGLUT1 and VGLUT2 are co-location in the CA1 and CA3 region of the hippocampus (83). Neale et al. concluded that VGLUT inhibitors can regulate glutamatergic synaptic transmission in hippocampus (81). This may be important in the pathophysiology of neurological diseases and may represent the goal of developing new treatments to drug. In addition, a study proved that eliminating the VGLUT2-dependent glutamatergic transmission of parvalbumin-expressing neurons leads to deficits in locomotion (84). At the same time, deletion of VGLUT2 weakened the spatial learning and memory and synaptic plasticity in the hippocampus of mice (85). Some studies suggested that the synaptic responses of acute slice and autaptic cultured rat hippocampal neurons were significantly decreased in the VGLUT1 knock-out rat, suggesting that VGLUT1 was the main transporter subtype in this region (86, 87). Drug abuse will produce cue-induced drug seeking memory information stored in the hippocampus, which plays an important role in the reward system (88, 89). In addition, hippocampus is involved mediating reward-related learning (90). The concentration of glutamate in the hippocampus can affect the excitability of neurons. Therefore, VGLUTS are very important to maintain the balance of glutamic acid concentration in hippocampus. However, few studies have reported the role of VGLUTS in addiction, especially in hippocampus.

### The Role of GLT in Drug Addiction Induced by Different Kinds of Drug

Addictive drugs can generally be divided into stimulants and inhibitors. For example, cocaine belongs to stimulants. Sakae et al. proved that knocking out VGLUT3 in the NAc aggravated cocaine-induced self-administration (35). In addition, Reissner et al. showed that propentofylline (PPF) restored the expression of glutamate transporter in the NAc induced by cocaine (91). Upregulation of glutamate transporter attenuates cocaine-seeking behavior (80). Morphine is a kind of inhibitor drug targeting opioid receptor that can lead to addiction. Glutamate transporter is a crucial role in morphine dependence (13). Chronic morphine administration induced downregulation of glutamate transporter expression in the NAc (92). Besides, activation of glutamate transporter results in inhibiting morphine tolerance (13). In other word, drug addiction will result in the changes of glutamate transporter. These studies reveal that upregulation of glutamate transporter is a promising method for treating drug addiction. And it also suggests that glutamate transporter is involved in drug addiction induced by different kinds of Addictive drugs. However, the underlying mechanism is still unclear.

## Summary

**Figure 2:**
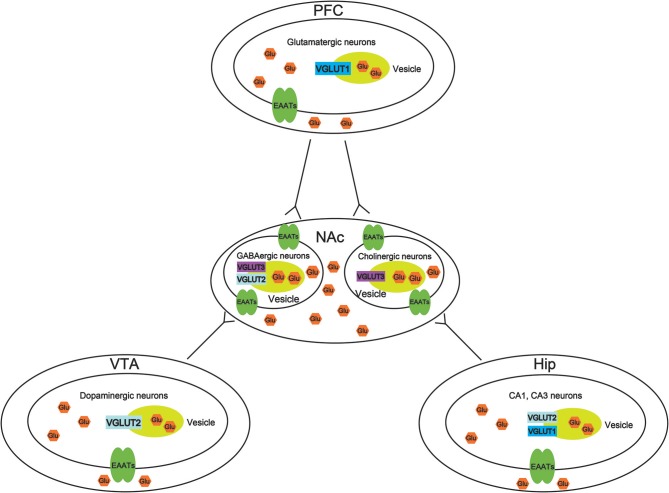
Molecular mechanisms of vesicular synergy. The role of different types of vesicular glutamate transporters (VGLUTS) in different brain regions in drug addiction. In the ventral tegmental area (VTA), VGLUT2 participates in the cooperative release of glutamate from dopaminergic neurons. In the nucleus accumbens (NAc), VGLUT2, and VGLUT3 participate in the cooperative release of glutamate from GABAergic neurons. VGLUT3 participates in the cooperative release of glutamate from cholinergic neurons. In the prefrontal cortex (PFC), VGLUT1 participates in the cooperative release of glutamatergic neurons. In the hippocampus (Hip), VGLUT1, and VGLUT2 participate in the cooperative release of glutamate from CA1 and CA3 neurons.

VGLUTS are very important to maintain the balance of the glutamate concentration in different brain regions, thus, increasing the potential mechanisms to treat drug abuse. However, there is little study to investigate the role of VGLUTS in drug addiction. In this review, we suggest that the role of different types of VGLUTS in different brain regions in drug addiction shown in Figure 2. As we know, PFC, VTA, NAc, and Hip are crucial to reward system. Therefore, it is necessary to understand that how VGLUTS influence or get involved in drug addiction. Previous studies have proved that glutamate neurotransmitter plays an important role in drug addiction. Therefore, the release of glutamate from different brain regions via glutamate transporter also play a crucial role. In addition, it is worth studying the role of glutamate transporter in other diseases of the central nervous system, such as Parkinson's disease and Alzheimer's disease. In this review, we also find that whether glutamate transporters can be targets for drug addiction remains to be studied (Tables 1, 2). Although Sakae et al. showed that knocking out VGLUT3 aggravates cocaine-induced self-administration (35), the other kinds of transporter in different brain region are still unclear. For example, by interfering or upregulating the expression of Glutamate transporter in different brain regions, and detecting addictive behaviors by conditioned place preference or self-administration. Beside that, the role of glutamate transporter in relapse and withdrawal are also worth exploring. For example, by interfering or upregulating the expression of VGLUT in different brain regions, and detecting addictive behaviors by conditioned place preference. Some studies have proved that VGLUTS expression level influence the rate and extent of synaptic vesicle filling, and the probability of synaptic vesicle release (87, 93–95). Glutamate is the main excitatory neurotransmitter in the human brain. It has been proved that long-term activation of the glutamate system can lead to nerve injury and cell death. Herman et al. proved that a low probability of release of glutamate when VGLUT expression levels were decreased (93). It is important that the abnormal level of these VGLUTS have been found in the pathophysiology of mental disorders. Therefore, in the future, VGLUTS may be a new target for treating drug addiction.

## Author Contributions

XL was responsible for the study concept and design. WW and YH drafted the manuscript. XL and FZ provided a critical revision of the manuscript for important intellectual content. All authors read and approved the final version.

### Conflict of Interest

The authors declare that the research was conducted in the absence of any commercial or financial relationships that could be construed as a potential conflict of interest.
